# Supplementary figures and images for: Reversal of cholestatic liver disease by the inhibition of sphingosine 1-phosphate receptor 2 signaling
Source: PeerJ. 2024 Jan 16;12:e16744. doi: 10.7717/peerj.16744 (PMC10798156; doi:10.7717/peerj.16744)

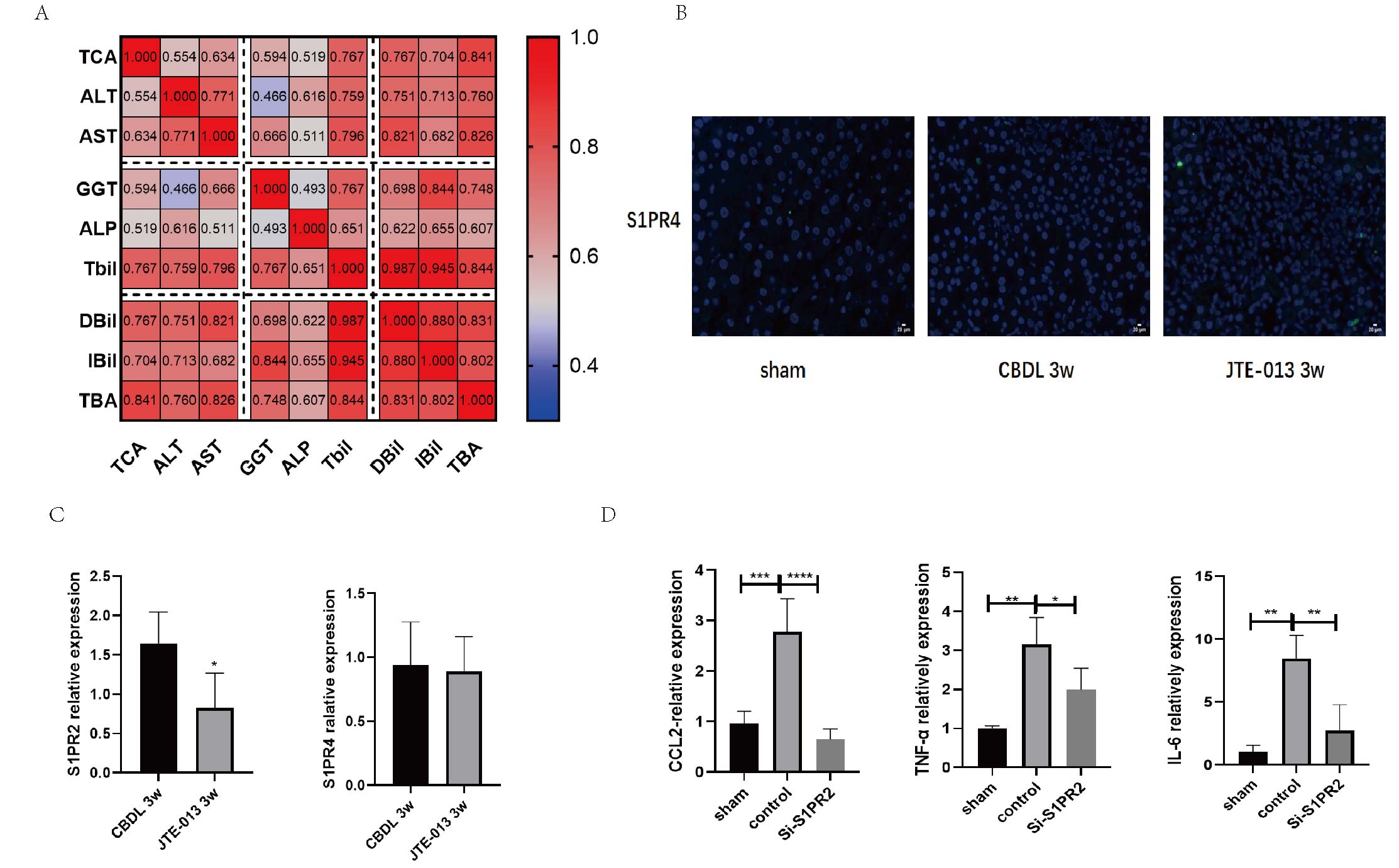

Supplement: Supplemental Information 2 — (A) The Spearman Correlation heatmap analysis between the liver function and taurocholic acid (TCA). (B) Immunofluorescence results show the level of S1PR4 in the liver was very low alleviate (scale bar = 20 um). (C) qPCR results showed that treatment of JTE-013 significantly decreased the level of S1PR2, however did not alter the level of S1PR4. (D) S1PR2 Si-RNA markedly inhibited pro-inflammation activation by TCA in BRL-3A (Rat Normal Hepatocytes Cells). [file peerj-12-16744-s002.jpg]

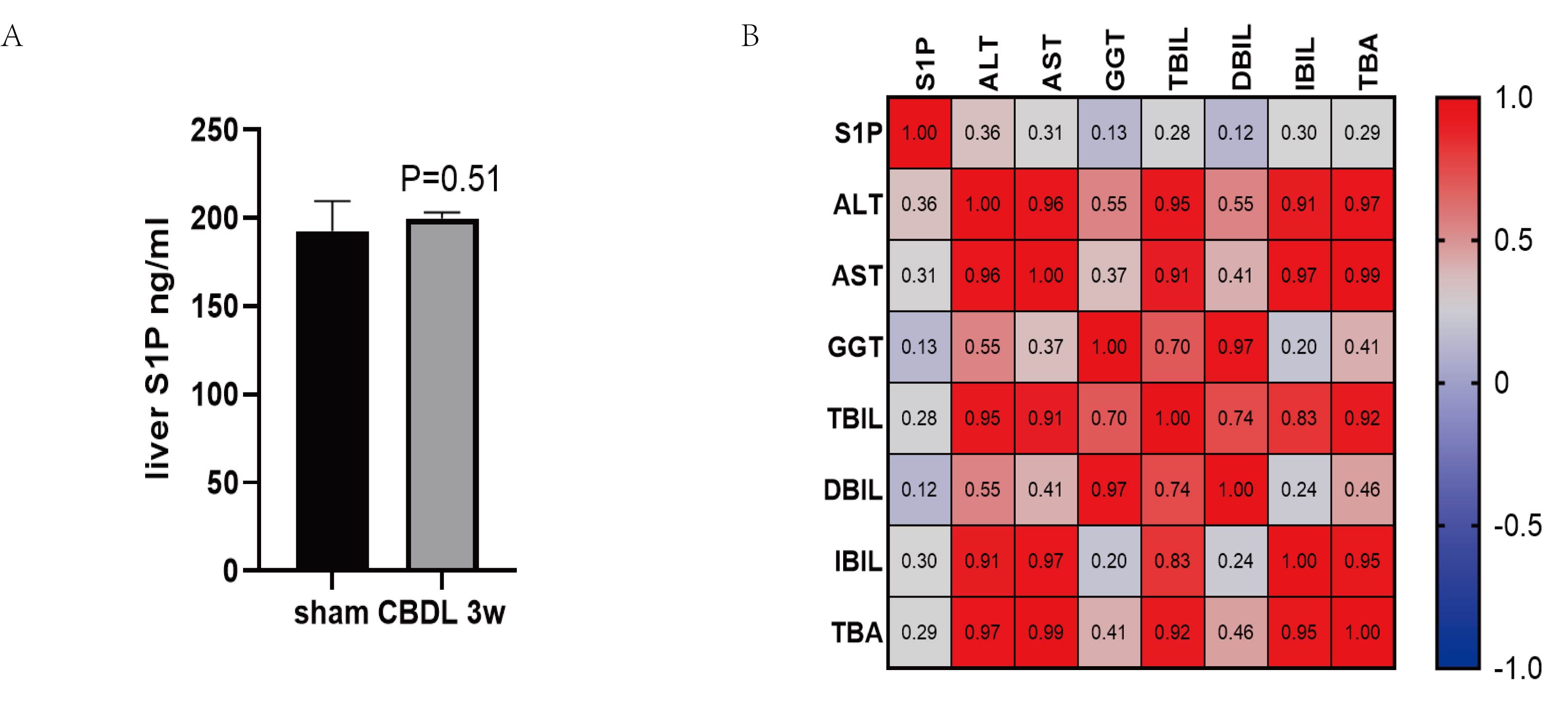

Supplement: Supplemental Information 3 — (A) The level of S1P in liver tissues was determined by its ELISA assay. (B) The Spearman Correlation heatmap analysis between the liver function and liver S1P. [file peerj-12-16744-s003.jpg]

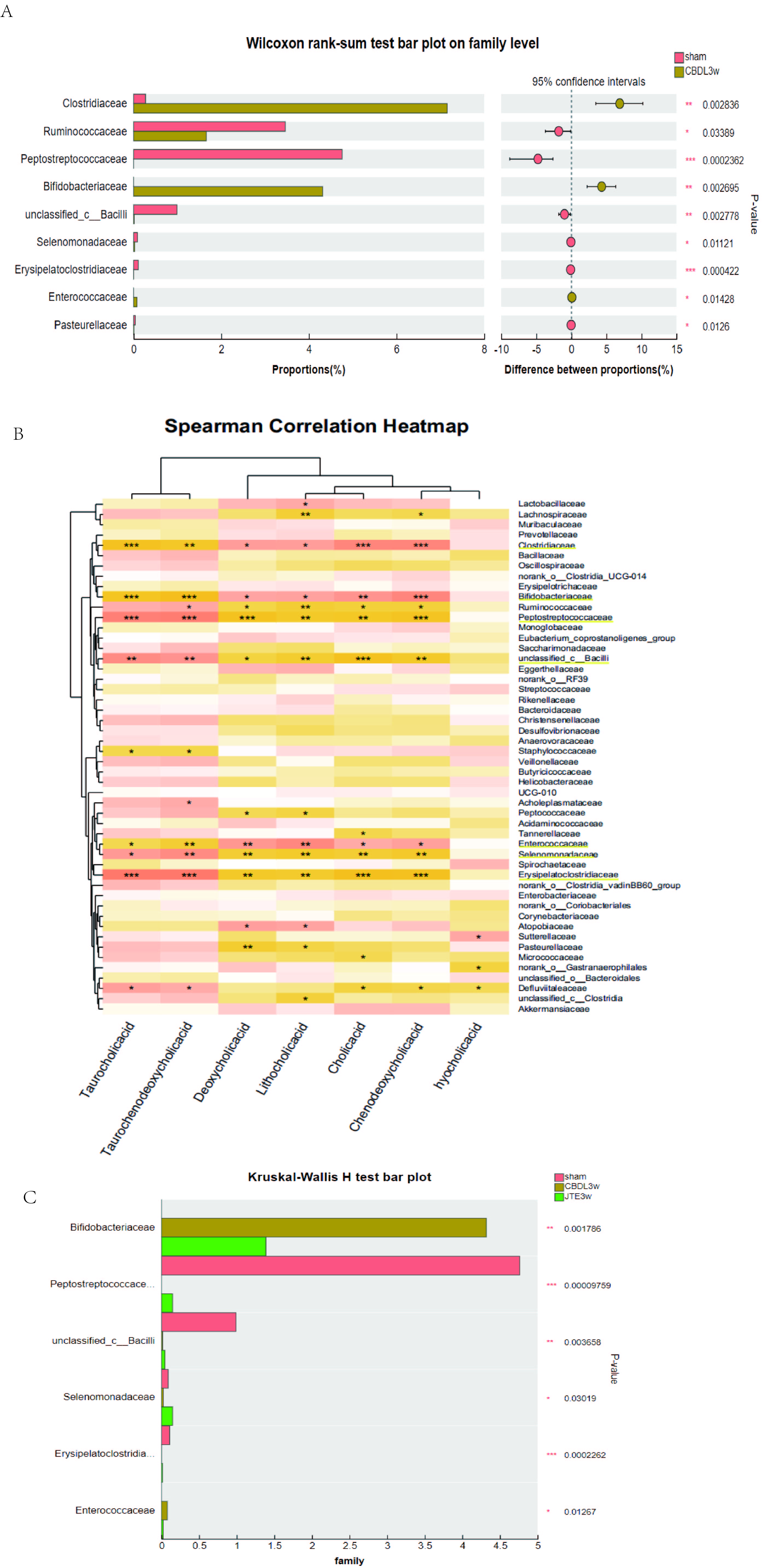

Supplement: Supplemental Information 4 — (A) Differential microbiota between the sham group and the CBDL 3w group at family level. (B) The Spearman Correlation heatmap analysis between the liver function and gut microbiota at family level. (C) Differential microbiota among the sham group, the CBDL 3w group and the JTE-013 3w group at family level. [file peerj-12-16744-s004.jpg]

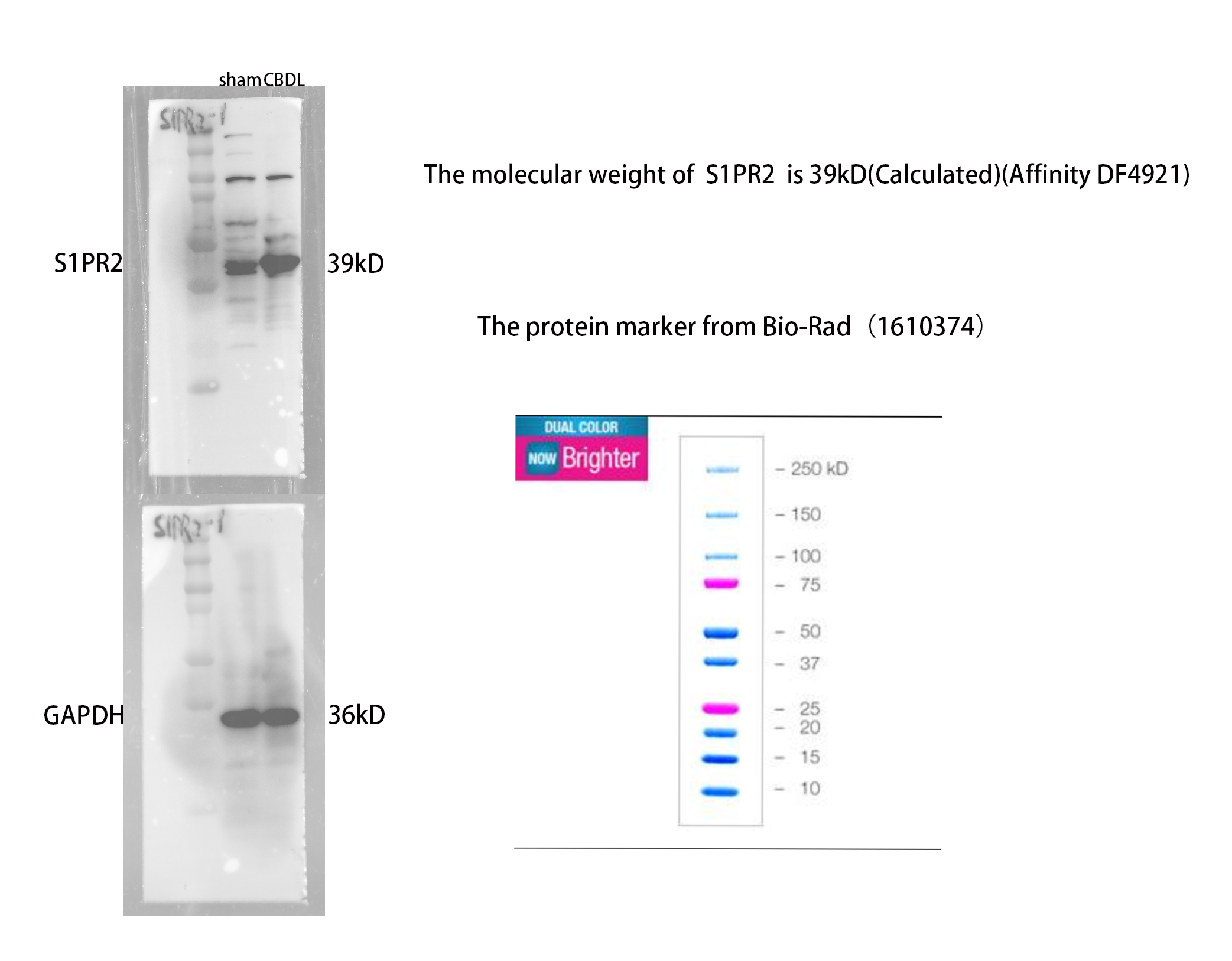

Supplement: Supplemental Information 6 [file peerj-12-16744-s006.png]

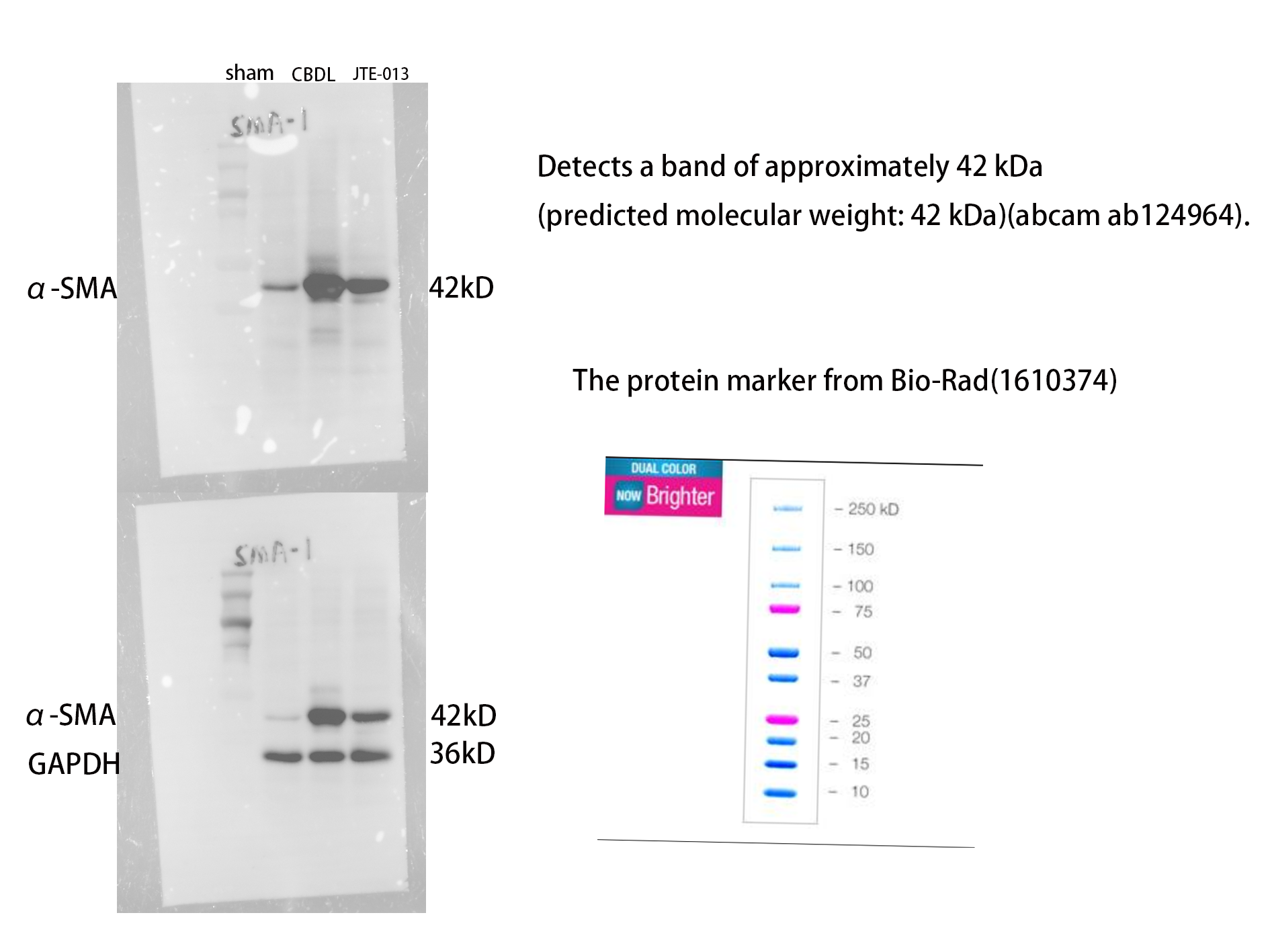

Supplement: Supplemental Information 7 [file peerj-12-16744-s007.png]

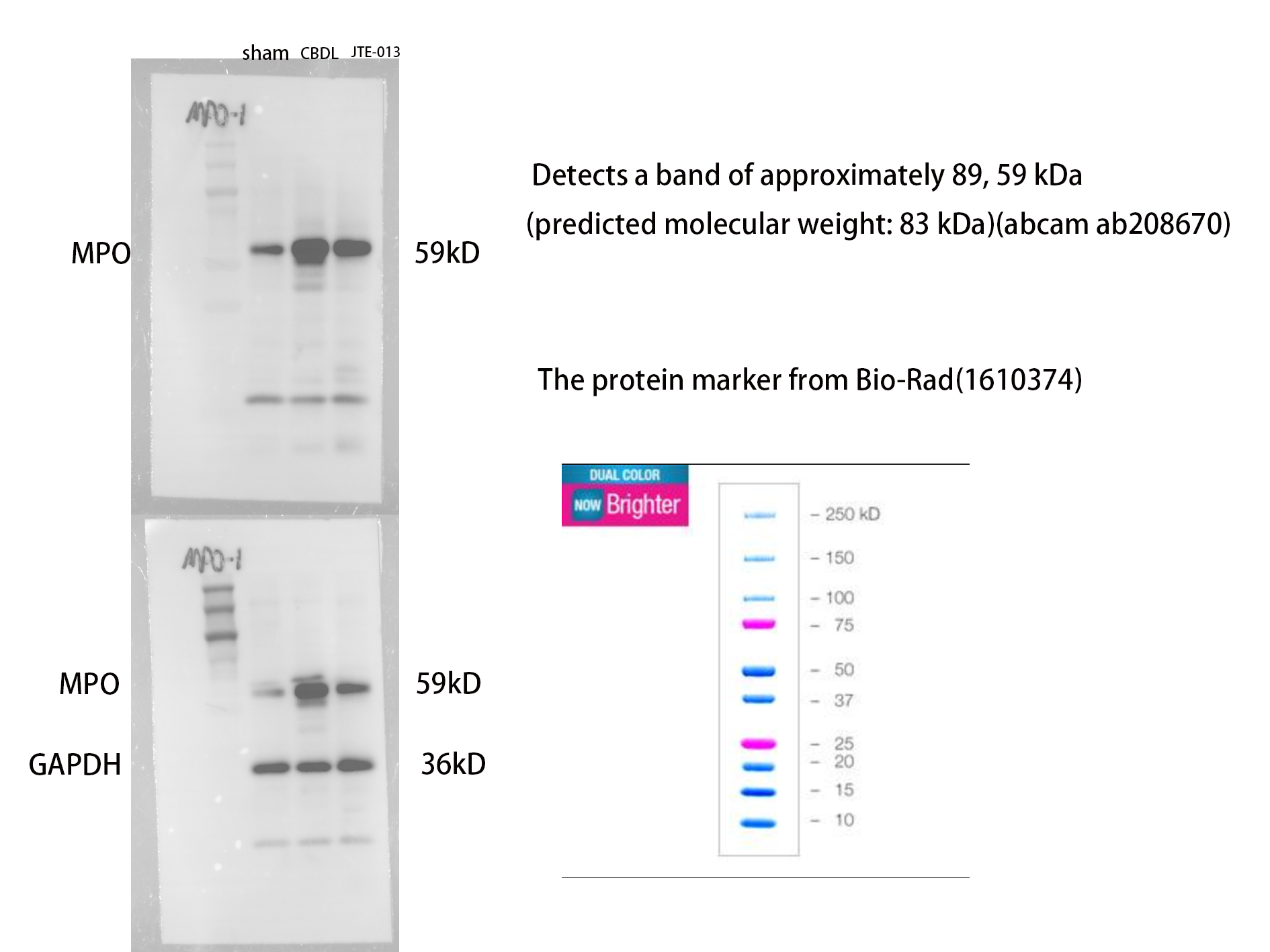

Supplement: Supplemental Information 8 [file peerj-12-16744-s008.png]

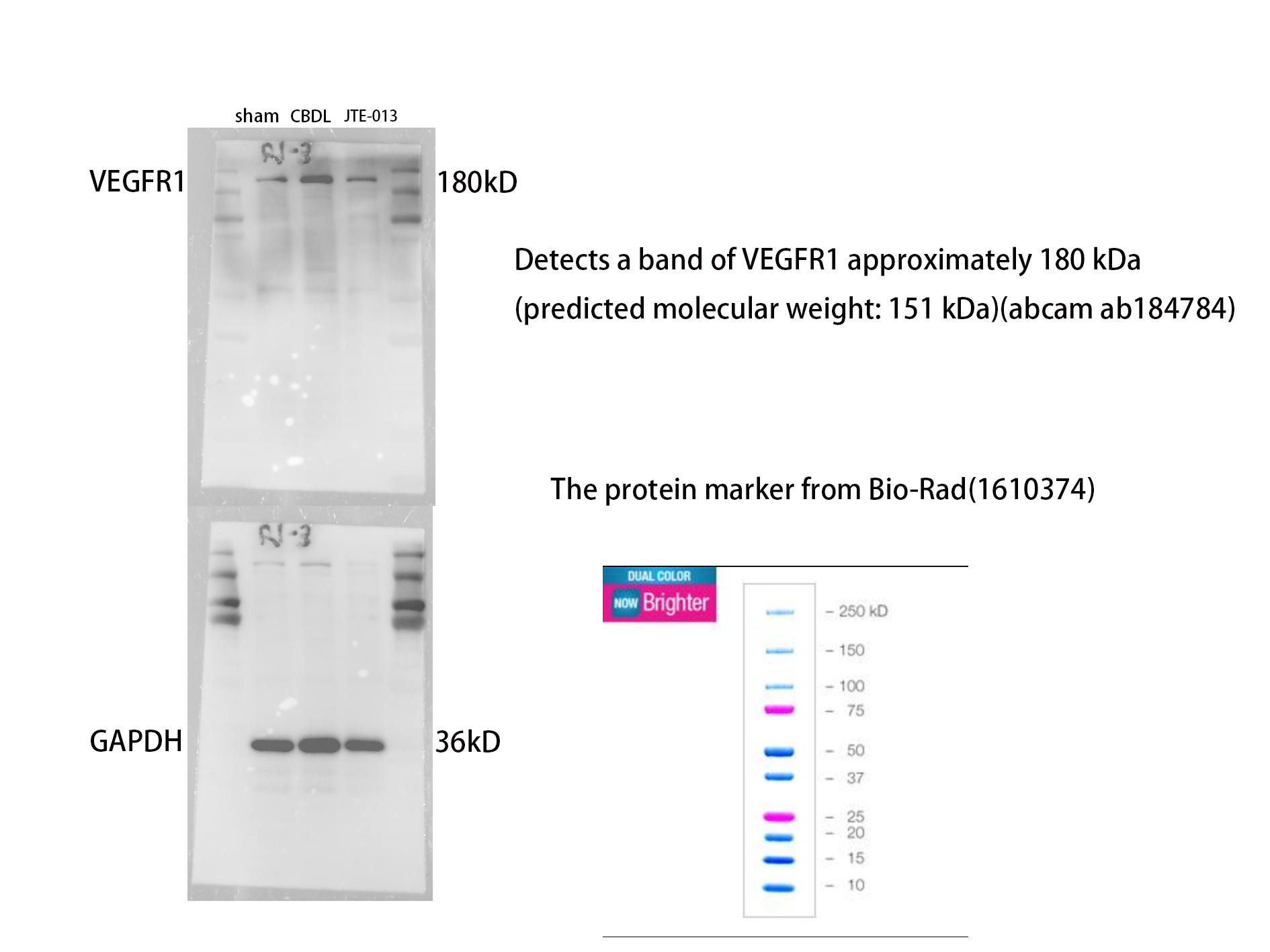

Supplement: Supplemental Information 9 [file peerj-12-16744-s009.png]
